# Does PI-ME/CFS recall post-COVID (PASC) syndrome?

**DOI:** 10.1016/j.virusres.2024.199393

**Published:** 2024-05-18

**Authors:** Salvatore Chirumbolo, Marianno Franzini, Umberto Tirelli

**Affiliations:** a Department of Engineering for Innovation Medicine, University of Verona, Italy; b Italian Scientific Society of Oxygen-Ozone Therapy (SIOOT)-High Master School of Oxygen Ozone Therapy, University of Pavia, Italy; c Tirelli Medical Group, Pordenone and Former Director Oncology, Aviano Cancer Center, Aviano (PN), Italy

ME/CFS (Myalgic Encephalomyelitis/Chronic Fatigue Syndrome) is a multifaceted condition characterized by persistent fatigue and a spectrum of other symptoms. Although its etiology remains elusive, theories implicate immune dysfunction, neuroendocrine abnormalities, among other factors. Of late, post-infectious triggers akin to those observed in post-COVID syndrome have piqued interest. Investigations underscore parallels between ME/CFS and post-COVID, spotlighting the intricate interplay of the gut microbiome-immune-brain axis, which may underpin shared symptoms. Notably, despite these commonalities, statistical analyses delineate distinctions between ME/CFS and post-COVID. Both conditions potentially involve disruptions in IGF-1 consequent to gut microbiome perturbations, potentially initiated by immune dysregulation. Consequently, adjunctive treatments aimed at modulating immunity hold promise for ameliorating symptoms in both cohorts. Overall, ME/CFS and post-COVID represent intricate syndromes with both shared and distinct features, possibly stemming from dysregulated immune responses and interactions within the gut microbiome.

For example, a recent contribution by Brian Walitt et al., from the Center for Human Immunology, Autoimmunity, and Inflammation (CHI) Consortium; Avindra Nath in Bethesda (MA), provided new hypotheses about the etiopathogenesis of post-infectious myalgic encephalomyelitis/chronic fatigue syndrome (PI-ME/CFS), which elicited some concerning issues we previously addressed about post-COVID (Walitt et al., 2024; Tirelli et al., 2023). Walitt and coworkers assessed that all PI-ME/CFS participants showed a typical set of symptoms, namely fatigue, physical symptoms and a decreased functional ability, exceeding at least 1.5–2.0 standard deviations in the worsening of the same symptoms in the general population (Walitt et al., 2024). Interestingly, fatigue, physical symptoms and a decrease in functional ability, are typical symptoms of the post-COVID syndrome (Abdel-Gawad et al., 2022; Oronsky et al., 2023).

Persistence of symptoms comparable to PI-ME/CFS in post-COVID may be caused even by persistence of SARS-CoV2 virus in the short term, although a recent meta-analysis failed in fully supporting this assumption, due to the lack of comparative group without a post-COVID symptomatology (C Fernández-de-Las-Peñas et al., 2024).

Moreover, some authors related the persistence of symptoms in post-COVID to the presence of auto-antibodies, as persistently positive anti-nucleus antibodies (ANAs) at 12 months post-COVID were associated with inflammation and long-lasting symptoms in post-COVID affected subjects (Son et al., 2023).

Following Walitt et al.’s paper, Anthony L. Komaroff commented the evidence reported by Walitt et al., and reminded readers that the complexity of ME/CFS in its involving brain, immunity, the gut microbiome brain axis (GMBA) and circulation, has been known for forty years, and confirmed, only few days after our report (Tirelli et al., 2023), that ME/CFS and post-COVID share similar symptoms and biological abnormalities (Komaroff and Lipkin, 2023).

The similarity between ME/CFS and post-COVID, sometimes known as long-COVID or Post-Acute Sequelae of COVID-19 (PASC) cannot be simply related to a shared symptomology, fatigue, cognition impairment, brain fog, muscle weakness, digestive issues, and so on, but they may be, as post-infected chronic syndromes (Walitt et al., 2024), a complex puzzle of an immune and GMBA pathology, as suggested by recent evidence (Walitt et al., 2024; Gentilotti et al., 2023; Righi et al., 2024).

This should allow clinicians to better elucidate the critical issues of reading symptoms as whether suggesting for the onset of a ME/CFS or a post-COVID, in present times. For example, some authors investigated ME/CFS before the occurrence of SARS-CoV2 pandemic and in this sense dismissed the problem (Walitt et al., 2024).

In the case of COVID-19, preventive measures such as mass vaccination and the use of SARS-CoV2 antivirals, might have reduced drastically the epidemiological prevalence and social impact of post-COVID (Notarte et al., 2022; C Fernández-de-Las-Peñas et al., 2024). A recent systematic review held on 2584 investigations, 11 peer-reviewed and six pre-print studies, rated a methodological quality of 82 % (*n* = 14/17) studies and only six of them, encompassing about 17,256,654 individuals, were selected as examining the impact of vaccination before the acute phase of SARS-CoV-2 infection. The report assessed that vaccination was associated with reduced risks or odds of long-COVID and that two doses were significantly more effective than one (Notarte et al., 2022).

A likelihood statistical test for ME/CFS and post-COVID was evaluated by selecting more than 400 samples from the existing literature in Pubmed/Medline, Embase, Scopus and WoS, and taking into account that 385 samples corresponded to the sample size for a margin of error lower than 5 % (CI = 95 %). The likelihood test was performed as ME/CFS (positive) matched with PASC (negative), within an error of 6.29 %. The likelihood ratios are very close to 1.00 for most of the symptoms shared by the two different pathologies, with the exception of 4/12 (33.3 %). The logistic regression allows to assess that, despite the great deal of shared symptoms between ME/CFS and PASC, the pathologies are different at *p* = 0.0020 (Fig. 1).

**Fig. 1:**
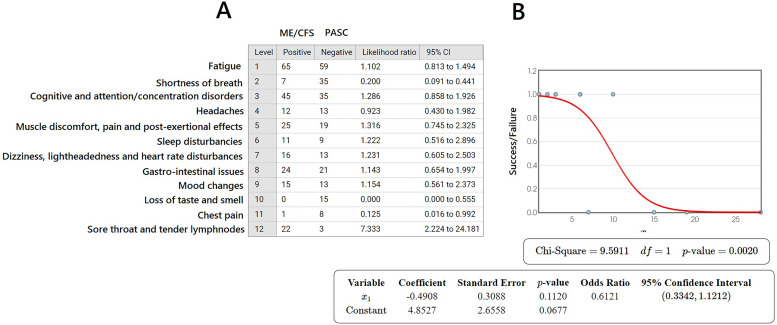
A) Table of likelihoods ratio calculations considering ME/CFS as the reference pathology (Positive) vs PASC (Negative) on the basis of 243 symptoms for each pathology, collected into 12 groups (on 490 samples retrieved from the current literature and from our clinical data). B) Logistic regression plot made according to (Sperandei, 2014) on data listed in A). For explanation see the text. Stata v.12 and SPSS v. 24.0.

Notwithstanding, it is of utmost importance to assess that for example fatigue following pneumonia, usually with chest or respiratory symptoms, are typical features of a post-COVID manifestation and that prolonged deficits in the pulmonary functions can lead to a symptomatology very akin to ME/CFS, at least for some particular cases. Anyway, the underlying hypothesis that join together ME/CFS with PASC may be associated with a severe impairment in GMBA. Walitt et al. reported on how much the gut microbiome is involved in the etiopathogenesis of PI-ME/CFS (Walitt et al., 2024). Interestingly, the authors excluded any psychiatric impact on PI-ME/CFS, yet recent suggestions shed a light on the paramount role exerted by an impaired GMBA in the post-COVID syndrome (Gentilotti et al., 2023; Righi et al., 2024). Impairments in the GMBA lead to mood disorders, anxiety and depressive behaviours (Grau-Del Valle et al., 2023), so, although we cannot confuse ME/CFS with the simplest psychiatric panel, the evidence reported by Walitt et al. gives an insightful focus on the etiopathogenesis of PI-ME/CFS and its purported similarity with post-COVID (Walitt et al., 2024).

Which is the truth? Are we addressing a new kind of complex and systemic syndrome regarding the relationship among gut, microbiome, immunity and brain?

The landscape in which PI-ME/CFS is described by some authors (Walitt et al., 2024), would suggest that the leading cause of the complex symptomatology characterizing PI-ME/CFS is a severe breakdown in the role of the GMBA in the body. This consideration can be put forward for PI-ME/CFS as well as for the post-COVID syndrome (Gentilotti et al., 2023; Righi et al., 2024).

Once SARS-CoV2 has infected its host and an immune thromboembolic disorder occurs (Bonaventura et al., 2021), endothelial dysfunction can exacerbate the impact of COVID-19 (Wu et al., 2024) and endothelia disorders are also a causative factor of fatigue in ME/CFS (Sandvik et al., 2023). Any microbial disruption leads to cerebral endothelial dysfunction (Rustia et al., 2021) and a close cross-talk between endothelia (microvasculature, circulation) and gut microbiome has been recently reported, involving the role of IGF-1R (Bouman Chen and Kaur Malhi, 2021).

The role of IGF-1 may be crucial.

For example, in children with inflammatory bowel syndrome (IBS) a lowered level of IGF-1 causes fatigue symptoms and a poor quality of life in these patients (Lucia Casadonte et al., 2018). Interestingly, fatigue observed in PI-ME/CFS did not depend on physiological issues such as resting energy expenditure, tissue oxygenation, muscle fibers composition, body composition, mechanical efficiency or so on, yet, once PI-ME/CFS patients undertook any physical task, they showed substantial differences with healthy volunteers (Walitt et al., 2024). As it is well known, IGF-1 maintains muscle mass and strength, becoming an important biomarker in physical exercise and cognition in the elderly (Stein et al., 2018).

Do PI-ME/CFS and post-COVID syndrome (Ilias et al., 2021) share an IGF-1 impairment due to gut microbiome disruption? And if so, what is the leading cause of the GMBA damage?

As we are referring to complex syndromes caused by infections, we are persuaded that impairment in the immune surveillance and tolerance might be the trigger factor of the gut microbiome damage, due to the crucial interplay between gut immunity and gut microbiota (Zheng et al., 2020).

The use of adjunct medical treatments with oxygen-ozone (U Tirelli et al., 2021; U Tirelli et al., 2021) to modulate immunity, in order to re-establish the correct cross-talk between the gut immune system and the gut microbiome, may be particularly promising in providing substantial relief in ME/CFS and in post-COVID symptomatology as well.

Both syndromes might be considered as complex pathologies triggered by an impaired immune cross talk with the GMBA following an infectious event. The scenario described to date allows researchers to make comparisons between PI-ME/CFS and post-COVID.

## CRediT authorship contribution statement

**Salvatore Chirumbolo:** Writing – review & editing, Writing – original draft, Visualization, Validation, Supervision, Methodology, Investigation, Formal analysis, Data curation, Conceptualization. **Marianno Franzini:** Data curation, Formal analysis, Software, Supervision, Validation, Writing – review & editing. **Umberto Tirelli:** Writing – review & editing, Visualization, Validation, Supervision, Conceptualization.

## Declaration of competing interest

The authors declare that they have no known competing financial interests or personal relationships that could have appeared to influence the work reported in this paper.

## Data Availability

Data will be made available on request.
